# PPR proteins in plants: roles, mechanisms, and prospects for rice research

**DOI:** 10.3389/fpls.2024.1416742

**Published:** 2024-06-27

**Authors:** Lingzhi Meng, Mengxue Du, Taotao Zhu, Gang Li, Yi Ding, Qiang Zhang

**Affiliations:** ^1^ College of Agricultural Science and Engineering, Liaocheng University, Liaocheng, China; ^2^ National Nanfan Research Institute, Chinese Academy of Agricultural Sciences, Sanya, China; ^3^ State Key Laboratory of Rice Biology and Breeding, China National Rice Research Institute, Hangzhou, China

**Keywords:** PPR proteins, RNA metabolism, chloroplast, mitochondria, stress response

## Abstract

Pentatricopeptide repeat (PPR) proteins constitute one of the largest protein families in land plants, with over 300 members in various species. Nearly all PPR proteins are nuclear-encoded and targeted to the chloroplast and mitochondria, modulating organellar gene expression by participating in RNA metabolism, including mRNA stability, RNA editing, RNA splicing, and translation initiation. Organelle RNA metabolism significantly influences chloroplast and mitochondria functions, impacting plant photosynthesis, respiration, and environmental responses. Over the past decades, PPR proteins have emerged as a research focus in molecular biology due to their diverse roles throughout plant life. This review summarizes recent progress in understanding the roles and molecular mechanisms of PPR proteins, emphasizing their functions in fertility, abiotic and biotic stress, grain quality, and chloroplast development in rice. Furthermore, we discuss prospects for PPR family research in rice, aiming to provide a theoretical foundation for future investigations and applications.

## 1 Introduction

PPR proteins is one of the largest protein families in higher plants, which are characterized by 2–30 tandemly arranged repeats. Each repeat consists of degenerated 30–40 amino acid PPR motifs (Qin et al., 2021). Each PPR motif forms a hairpin structure with a pair of anti-paralleled α-helixes, recognizing nucleotides in RNA through interactions with residues at position 6′ and 1′ of the motif (Barkan and Small, 2014). These proteins have been identified in various plant species, such as *Arabidopsis* (Lurin et al., 2004), foxtail millet (Xing et al., 2018), poplar (Liu et al., 2016), and maize (Chen et al., 2018a, b), with 441, 486, 626, and 491 members, respectively. PPR proteins are classified into two main subfamilies, P and PPR-like (PLS), based on PPR motif architectures (Cheng et al., 2016). The ancestral P subfamily proteins contain canonical 35 amino acid P (PPR) motifs. PLS subfamily proteins have P-, L- (long PPR, 35 or 36 amino acids), and S- (short PPR, 31 or 32 amino acids) tandem repeats. The PLS subfamily is further divided into PLS, E+, E, and DYW subclasses according to the characteristic C-terminal domain (Lurin et al., 2004; Li et al., 2021).

**Figure 1:**
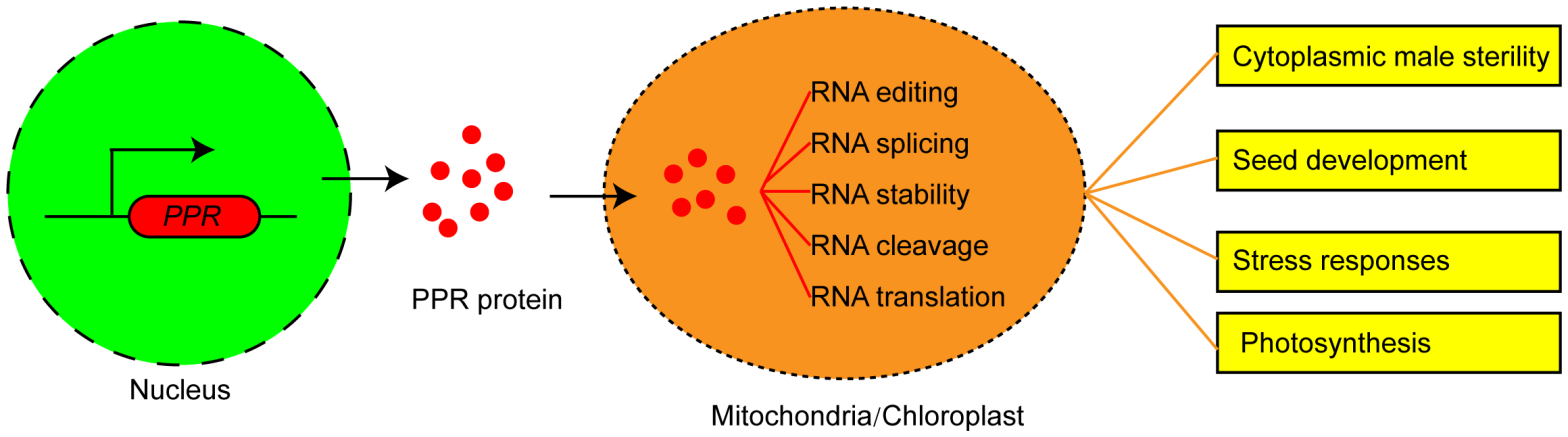
The functions and molecular mechanism of PPR proteins in plants. PPR proteins are encoded by nuclear genes, translated in the cytoplasm, and then imported into mitochondrion or chloroplast to mediate multiple steps of RNA processing and determine the plant phenotype.

Eukaryotic nuclear genome encodes PPR proteins, which are primarily post-translationally transported to mitochondria or chloroplast (Barkan et al., 2012; Cheng et al., 2016). PPR proteins play critical roles in the plant organelle RNA processes. PPRs could not only act as site recognition factors but also bind to cis-elements specifically. The resultant PPR-RNA complex participates in RNA editing, RNA splicing, RNA stability, RNA cleavage, and RNA translation (**Figure 1**). P-type PPR proteins predominantly participate in organelle RNA transcript splicing, stability, and translation. PLS-type PPR proteins mainly function in mitochondrial and chloroplastic RNA editing (Gruttner et al., 2022). These two PPR protein types may exhibit partially overlapping functions in regulating RNA splicing and editing.

Extensive research has demonstrated the diverse functions of PPR proteins in plant growth and development, including cytoplasmic male sterility (CMS), seed development, photosynthesis, and responses to biotic and abiotic stresses (Li et al., 2021). Most chloroplast-localized PPRs are associated with photosynthesis, mutations of which result in photosynthetic defects, aberrant leaf development and decreased leaf pigmentation (Hao et al., 2019). Some mitochondrial-localized PPRs are involved in seed development. These PPRs mutation generally causes floury/defective endosperm and retarded growth (Yang et al., 2017; Wang et al., 2019). CMS is considered to be jointly regulated by mitochondrial genes and their corresponding restorer of fertility (*Rf*) genes. Most *Rf* genes belong to the PPR genes family, which can restore fertility by suppressing the production of mitochondrial CMS associated proteins (Li et al., 2021). RNA editing contributes to the adaptation of land plants to extreme temperature, UV, and oxidative stress. Increasing evidences have showed that biotic and abiotic stresses change the expression patterns of PPR genes. To date, many PPR genes have been reported to be associated with salt stress, drought stress, cold stress, and defense response (Jiang et al., 2015; Qiu et al., 2021).

**Figure 2:**
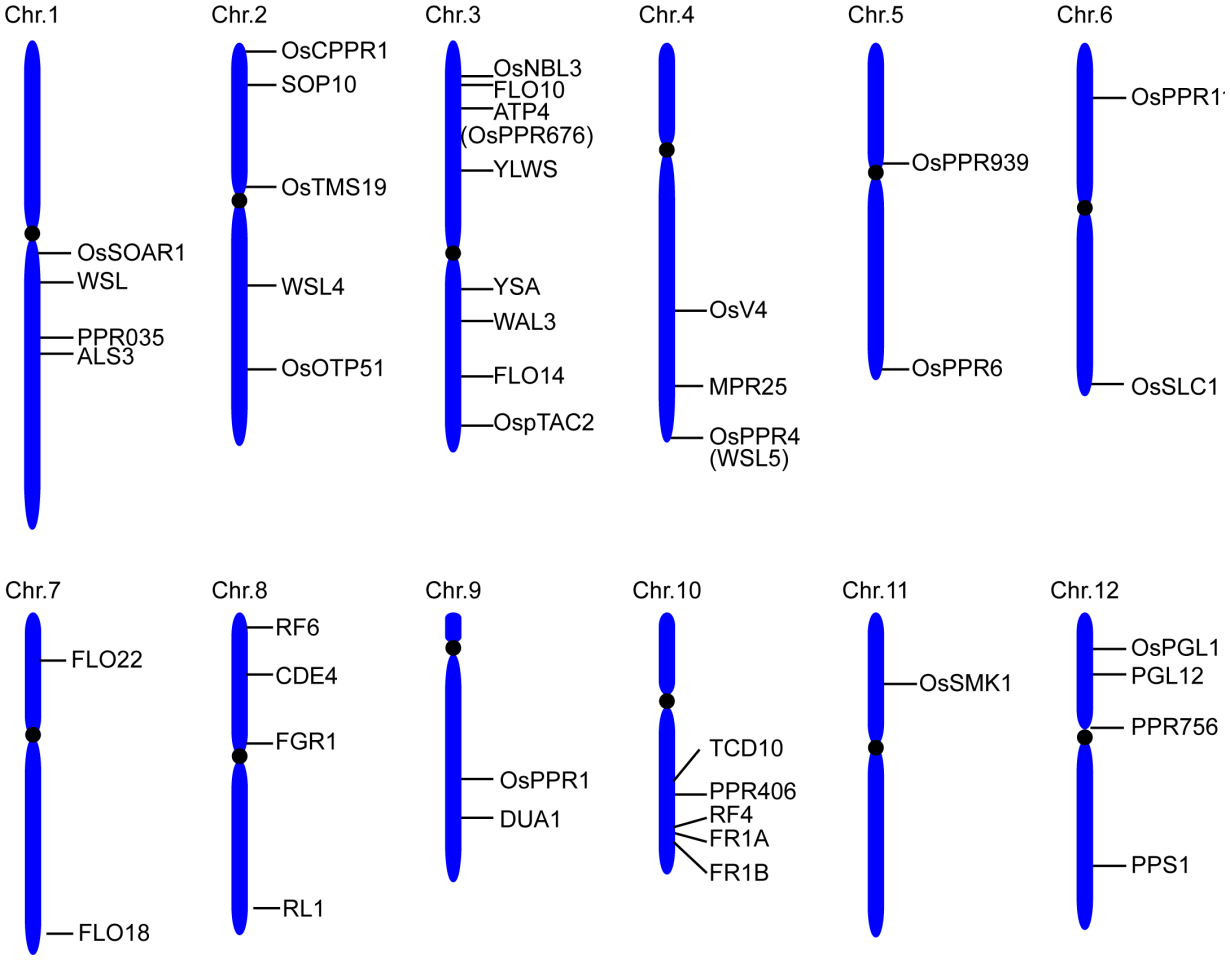
Distribution of the cloned PPR proteins on rice chromosomes.

**Figure 3:**
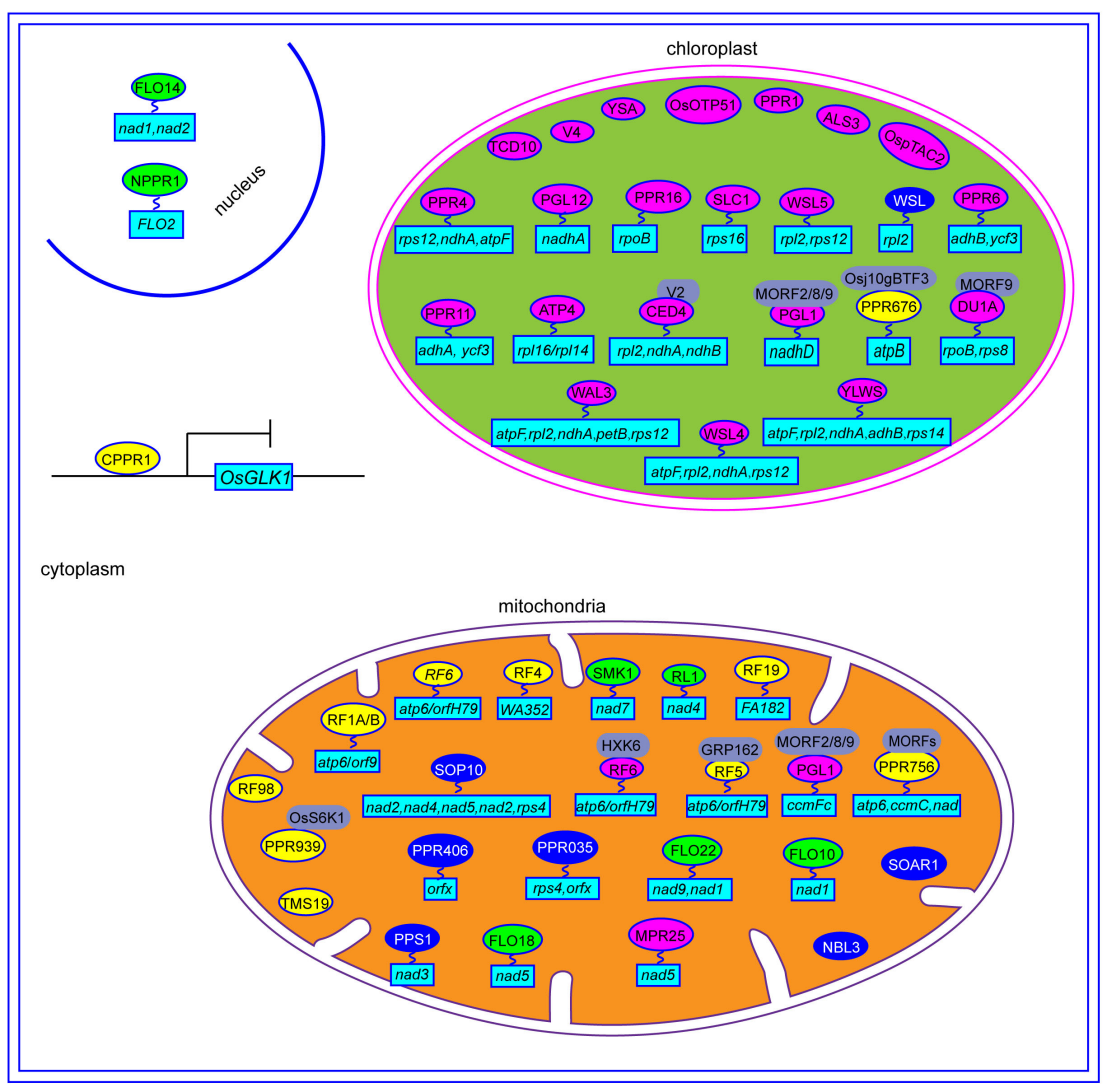
The regulation mechanisms of PPR proteins in rice. The yellow ovals represent PPR protein involved in CMS. The green ovals represent PPR protein involved in grain quality. The blue ovals represent PPR protein involved in stress responses. The red ovals represent PPR protein involved in chloroplast development. The gray ovals represent the interaction protein of PPR. The light blue rectangles represent the genes that are regulated by PPR proteins.

A total of 491 *PPR* genes were identified in the rice genome, distributed across all 12 rice chromosomes. Predictions revealed that most PPR proteins were targeted to either mitochondria (54%) or plastid (28%). Among them, 245 and 246 PPR proteins belong to the PLS and P subfamilies, respectively. Structural analysis indicated that in rice, 319 *PPR* genes lack introns, 79 genes contain one intron, and 93 genes harbor more than one intron (Chen et al., 2018a). To date, at least 48 PPR genes distributed in all 12 rice chromosomes, have been cloned and identified in rice (**Figure 2**, **Table 1**). Among the 48 PPR genes, most of them are confirmed to regulate rice development by directly or indirectly influencing the RNA splicing, RNA editing, RNA cleavage, RNA degradation, and RNA translation. Most cloned PPR proteins are targeted to mitochondria and chloroplast, with only a few being targeted to the nucleus and cytoplasm (**Figure 3**). This review summarizes the recent progress on PPR proteins in rice and discusses their roles and molecular mechanisms in fertility, stress response, grain quality, and chloroplast development.

**Table 1:** List of PPR proteins cloned and identified in rice.

Protein name	Accession number	Protein category	Subcellular localization	References
RF1A	LOC_Os10g35436	P	mitochondria	Wang et al., 2006
RF1B	LOC_Os10g35640	P	mitochondria	Wang et al., 2006
RF4	LOC_Os10g35240	P	mitochondria	Tang et al., 2014
RF5	AB179840	—	mitochondria	Hu et al., 2012
RF6	LOC_Os08g01870	P	mitochondria	Huang et al., 2015
OsRF19	UVZ00832	—	mitochondria	Jiang et al., 2022
RF98	—	—	—	Igarashi et al., 2016
OsPPR939	LOC_Os05g19390	P	mitochondria	Zheng et al., 2021
PPR756	LOC_Os12g19260	PLS	mitochondria	Zhang et al., 2020a
OsTMS19	LOC_Os02g21580	P	mitochondria	Zhou et al., 2024
OsPPR676	LOC_Os03g11670	PPR-SMR	plastid	Liu et al., 2017
OsCPPR1	LOC_Os02g02020	P	cytoplasm	Zheng et al., 2022
PPS1	LOC_Os12g36620	PLS	mitochondria	Xiao et al., 2018b
PPR035	LOC_Os01g46230	PLS	mitochondria	Luo et al., 2022
PPR406	LOC_Os10g30760	PLS	mitochondria	Luo et al., 2022
SOP10	LOC_Os02g07050	PLS	mitochondria	Zu et al., 2023
*OsSOAR1*	LOC_Os01g32170	SOAR1-like	—	Lu et al., 2022
WSL	LOC_Os01g37870	P	chloroplast	Tan et al., 2014
OsNBL3	LOC_Os03g06370	P	mitochondria	Qiu et al., 2021
*FLO10*	LOC_Os03g07220	P	mitochondria	Wu et al., 2019
*FLO18*	LOC_Os07g48850	P	mitochondria	Yu et al., 2021
FLO22	LOC_Os07g08180	P	mitochondria	Yang et al., 2023
*Os_SMK1*	LOC_Os11g10740	PLS	mitochondria	Li et al., 2014
*RL1*	LOC_Os08g41380	PLS	mitochondria	Wu et al., 2020
FGR1	LOC_Os08g19310	P	nuclear	Hao et al., 2019
FLO14	LOC_Os03g51840	P	nuclear	Xue et al., 2019
*OsPPR1*	LOC_Os09g24680	PLS	chloroplast	Gothandam et al., 2005
WAL3	LOC_Os03g44210	PLS	chloroplast	Lv et al., 2022
*YLWS*	LOC_Os03g1965	P	chloroplast	Lan et al., 2023
ALS3	LOC_Os01g48380	P	chloroplast	Lin et al., 2015
OSOTP51	LOC_Os02g47360	P	chloroplast	Ye et al., 2012
WSL4	LOC_Os02g35750	P	chloroplast	Wang et al., 2017
PGL12	LOC_Os12g10184	PLS	chloroplast	Chen et al., 2019
OsPPR16	—	PLS	chloroplast	Huang et al., 2020
YSA	LOC_Os03g40020	P	chloroplast	Su et al., 2012
OsPPR4	LOC_Os4g58780	P	chloroplast	Lee et al., 2019
OsPPR11	LOC_Os06g09880	P	chloroplast	Zhang et al., 2023
OspTAC2	LOC_Os03g60910	PPR-SMR	chloroplast	Wang et al., 2016
OsPPR6	LOC_Os05g49920	PLS	chloroplast	Tang et al., 2017
OsSLC1	LOC_Os06g49670	P	chloroplast	Lv et al., 2020
MPR25	LOC_Os04g51350	PLS	mitochondria	Toda et al., 2012
OsPGL1	LOC_Os12g06650	PLS	mitochondria chloroplast	Xiao et al., 2018b
TCD10	LOC_Os10g28600	P	chloroplast	Wu et al., 2016
OsV4	LOC_Os04g39970	P	chloroplast	Gong et al., 2014
ATP4	LOC_Os03g11670	PPR-SMR	chloroplast	Zhang et al., 2020b
WSL5	LOC_Os04g58780	P	chloroplast	Liu et al., 2018
CDE4	LOC_Os08g09270	P	chloroplast	Liu et al., 2021
DUA1	LOC_Os09g29825	PLS	chloroplast	Cui et al., 2019

“—”, represents the subcellular localization of the PPR proteins is not confirmed.

## 2 Functions of PPR proteins in rice fertility

**Figure 4:**
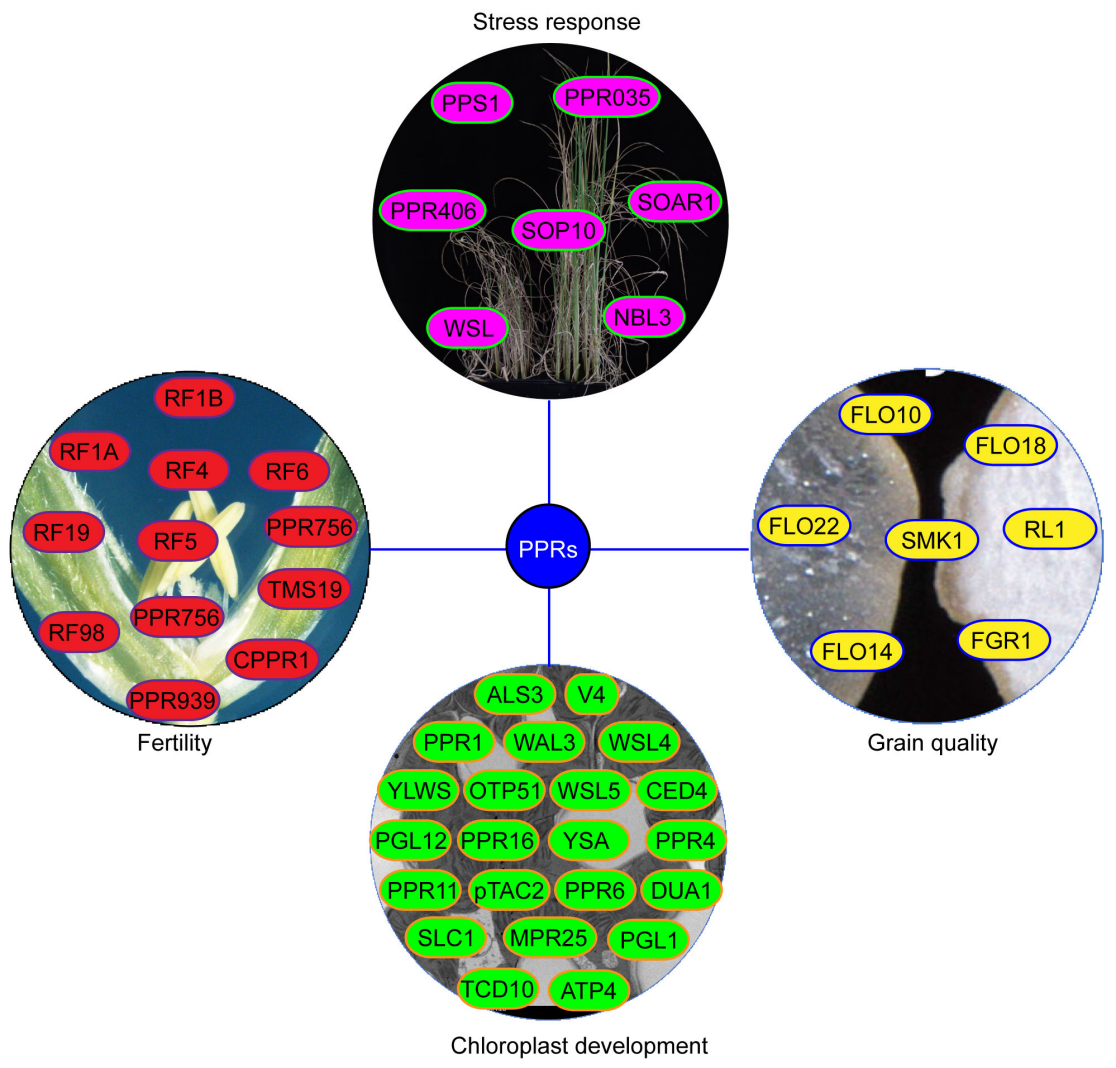
The functions of PPR proteins in rice. The yellow proteins represent PPR protein involved in grain quality. The green proteins represent PPR protein involved in chloroplast development. The red proteins represent PPR protein involved in CMS. The light red proteins represent PPR protein involved in stress responses.

CMS is a prevalent maternally inherited trait in flowering plants, characterized by female fertility and abortive pollen (Liu et al., 2017). Mitochondrial genes and their corresponding nuclear-encoded restorer of fertility (*Rf*) genes reportedly control CMS by regulating the expression of CMS-related genes in mitochondria (Wang et al., 2006). The CMS/Rf system is not only essential for hybrid seed production but also serves as an ideal genetic tool to investigate heterosis and mitochondrial-nuclear genetic interactions (Chen and Liu, 2014). To date, numerous rice *Rf* genes have been cloned and characterized, mostly belonging to PPR proteins (**Figure 4**). Rf1A and Rf1B, containing 19 and 11 PPR repeats, respectively, are mitochondrial and restore male sterility by inhibiting the cytotoxic ORF79 peptide production, which causes gametophytic male sterility. Rf1a and Rf1b restore male fertility via the cleavage and degradation of mitochondrial chimeric gene B-*atp6*/*orf79* mRNA, respectively (Wang et al., 2006). *Rf4*, encodes a mitochondrial P-subfamily protein with 19 PPR repeats, rescuing Wild-Abortive CMS (WA-CMS) by suppressing CMS-associated gene *WA352* transcription (Tang et al., 2014). *Rf5*, the rice fertility restoration gene of Hongliian CMS (HL-CMS), physically interacts with GRP162, which binds *atp6*-*orfH79* via an RNA recognition motif, thereby promoting the *atp6*-*orfH79* transcript processing (Hu et al., 2012). The interaction between the PPR proteins OsRF6 and hexokinase OsHXK6 separates the mitochondrial chimeric gene *atp6*-*orfH79* into two fragments, *atp6* and *orfH79*, to restore the fertility of HL-CMS lines (Huang et al., 2015). OsRF19 rescues male sterility of Fujian Abortive CMS (FA-CMS) by mediating the RNA cleavage of the chimeric gene *FA182*. *OsRf19* originates from a recent duplication in wild rice relatives and has a common ancestor with *OsRf1a*/*OsRf*5 (Jiang et al., 2022). *Rf98* (*PPR762*) is an essential fertility restorer gene for RT98-type CMS, which only restores partial fertility, thereby implying the presence of additional genes near the *Rf98* locus (Igarashi et al., 2016).

Besides the *Rf* genes, some genes play a role in regulating pollen development. For instance, OsPPR939 is crucial for splicing of mitochondrial *nad5* introns 1, 2, and 3. The OsPPR939 protein can be phosphorylated by OsS6K1, which facilitates its mitochondrial import. Its disruption results in growth retardation and pollen sterility (Zheng et al., 2021). PPR756 participates in editing mitochondrial genes *atp6*, *ccmC*, and *nad* and interacts with organellar RNA editing factors OsMORF1, OsMORF8–1, and OsMORF8–2 to form editosome complexes. Loss of *PPR756* function abolishes RNA editing of *atp6*, *ccmC*, and *nad*, thereby causing retarded growth and pollen sterility (Zhang et al., 2020a). A point mutation (T-A) in the *OsTMS19* exon is responsible for photo/thermo-sensitive genic male sterility. Excessive ROS accumulation in *ostms19* anthers leads to male sterility, whereas effective ROS scavenging restores fertility (Zhou et al., 2024).

The PPR proteins regulating rice fertility, as previously mentioned, are primarily targeted to the mitochondria. However, two other PPR-SMR proteins, OsPPR676 and OsCPPR1, are localized to the plastid and cytoplasm, respectively, while also being associated with pollen development. OsPPR676 interacts with Osj10gBTF3, a NAC protein involved in pollen development regulation. The *osppr676* mutant exhibits disrupted *atpB* mRNA translation, growth retardation, and partial pollen sterility (Liu et al., 2017). OsCPPR1 directly binds to single-stranded regions of *OsGLK1*, thereby regulating its transcription. In the *oscppr1* mutant, *OsGLK1* expression is significantly upregulated, which causes abnormal plastid development, prolonged tapetal programmed cell death, and tapetum degradation. Consequently, pollen fertility is significantly decreased (Zheng et al., 2022).

## 3 Functions of PPR proteins in biotic and abiotic stress responses

Multiple studies have demonstrated the involvement of numerous PPR proteins in rice’s response to biotic and abiotic stresses. Chen et al. (2018a) reported up-regulation of 75 and 73 *PPR* genes under salt and drought stresses, respectively, compared to control. In a separate study, Luo et al. (2022) analyzed *PPR* gene expression across different stress treatments and identified 16/81, 15/127, and 27/35 *PPR* genes that were upregulated/downregulated in response to osmotic, salt, and oxidative stress, respectively (Luo et al., 2022).

Several PPR proteins have been identified to play roles in the biotic and abiotic stresses. Except WSL, most PPR proteins are primarily localized in the mitochondria (**Figure 3**). PPS1, a PLS-type PPR protein, participates in C-to-U RNA editing of *nad3* transcripts by binding to cis-elements near its five conserved RNA-editing sites. *PPS1*-RNAi plants exhibit decreased editing efficiency, increased ROS accumulation, and heightened sensitivity to abiotic stresses such as salinity and ABA, when compared to WT (Xiao et al., 2018a, 2021). Both PPR035 and PPR406 are localized in mitochondria, with PPR035 affecting the editing efficiency of *rps4*-C926 and *orfx*-C406, while PPR406 affects the editing efficiency of *orfx-C355*. Although both *prp035* and *ppr406* mutants improve drought and salt stress resistance, their underlying molecular mechanisms remain unclear (Luo et al., 2022). Recent study showed that SOP10 affects splicing efficiency of *nad4* and *nad5* introns and RNA editing efficiency of *nad2*, *nad6*, and *rps4* transcripts in the mitochondria. Mutation of *SOP10* leads to mitochondrial complex I deficiency, which inhibits ROS production and enhances cold tolerance in *indica* rice varieties (Zu et al., 2023). The transgenic rice lines overexpressing *OsSOAR1* demonstrate enhanced salt tolerance during seedling growth, along with increased chlorophyll content and reduced ion leakage compared to WT (Lu et al., 2022).

Belonging to the P-subfamily of PPR proteins, WSL is localized in the chloroplast and is involved in chloroplast transcript *rpl2* splicing. In *wsl* mutants, PEP-dependent plastid gene expression is significantly down-regulated, and plastid rRNAs and translation products accumulate to very low levels compared to WT. Mutant *wsl* exhibits defective *rpl2* splicing, which generates a white striped phenotype in seedlings, and increases sensitivity to ABA and salinity stress (Tan et al., 2014).

Additionally, PPR proteins are implicated in biotic stress. *OsNBL3* encodes a mitochondrion-localized P-type PPR protein, with *nbl3* mutants displaying a lesion mimic phenotype, spontaneous cell death, enhanced resistance to *Magnaporthe oryzae* and *Xanthomonas oryzae* pv. *Oryzae*, and improved salt tolerance. OsNBL3 is involved in splicing mitochondrial gene *nad5* intron 4. Disruption of *OsNBL3* reportedly reduced complex I activity, increased alternative respiratory pathways, and damaged mitochondrial morphology (Qiu et al., 2021). These findings contribute to a better understanding of PPR genes and their functions in biotic and abiotic stresses. Therefore, we can use gene engineering or traditional hybridization concentrated on the PPR gene to improving rice adaptability.

## 4 Effects of PPR proteins on rice grain quality

Endosperm, the primary storage organ in cereal grains, significantly influences grain yield and quality. Several PPR proteins reportedly regulate endosperm development in rice (**Figure 4**). *FLO10* and *FLO18* encode mitochondrion-targeted P-type PPR proteins with 26 and 15 PPR motifs, respectively. Loss of *FLO10* (*WBG1*) disrupts splicing of mitochondrial *nad1* intron 1 and increases accumulation of *nad1* exon 1 and exons 2–5 precursors (Wu et al., 2019). Disruption of *FLO18* function impairs 5′-end processing of mitochondrial *nad5* mRNA (Yu et al., 2021). Mutants of both *FLO10* and *FLO18* exhibit reduced assembly and activity of mitochondrial complex I in the electron transport pathway, altering mitochondrial morphology and resulting in abnormal endosperm development characterized by smaller starch grains, reduced starch content, and abnormal aleurone cells (Wu et al., 2019; Yu et al., 2021; Wu et al., 2023). The mitochondrion-tagged P-type PPR protein FLO22 directly binds to the”GAAGUGGAAG”sequence of *nad1*, thereby influencing the splicing and editing efficiency of *nad1* and *nad9* mRNA, respectively. FLO22 interacts with DYW3, a DYW-type PPR protein, forming a complex that likely synergistically functions in mitochondrial RNA editing. Mutation of *FLO22* leads to alterations in complex I activity, respiration rate, mitochondrial morphology, and function, resulting in opaque, floury mature grains (Yang et al., 2023). Both *Os_SMK1* and *RL1* encode mitochondrion-targeted PPR-E subclass proteins, which are involved in C-U editing of *nad7–*836 and splicing of mitochondrial *nad4* intron 1, respectively. Mutants of both *Os_SMK1* and *RL1* display chalky endosperm (Li et al., 2014; Wu et al., 2020).

Contrary to earlier findings suggesting chloroplast or/and mitochondrial localization for most PPR proteins, the FGR1 (OsNPPR1) and FLO14 (OsNPPR3) proteins, from the P-subfamily PPR proteins, are instead nuclear-localized (Hao et al., 2019; Xue et al., 2019). OsNPPR1 directly binds to the”CUCAC” motif, whose mutation alters splicing of certain nuclear genes linked to mitochondrial functions, thereby causing intron retention. The *fgr1* mutant exhibits opaque endosperm with numerous smaller, single starch grains (SGs) and reduced amylose content (Hao et al., 2019). Furthermore, *FLO14* mutation reduces splicing efficiency of mitochondrial genome-encoded transcripts, *nad1–2* and *nad2.* The *flo14* mutant displays a chalky endosperm phenotype with immature, smaller, and more scattered starch granules and lower starch content compared to WT (Xue et al., 2019).

## 5 Roles of PPR proteins in rice chloroplast development

Chloroplasts, essential for plant growth and development, generate energy for respiration and other physiological processes by fixing carbon and releasing oxygen (Lan et al., 2023). As a semi-autonomous organelle with its own genome, chloroplasts development rely on coordinated expressions of both chloroplast and nuclear genes (Woodson and Chory, 2008). Proteins encoded by nuclear genes are transported into organelles to regulate chloroplast gene expression at various levels, including transcriptional and post-transcriptional mechanisms such as RNA splicing, editing, and translation (Xin et al., 2021). Notably, PPR proteins serve as vital cofactors in chloroplast development and gene regulation (**Figure 4**). The *OsPPR1* gene was the first rice *PPR* gene involving in chloroplast development in 2005. Transgenic plants expressing antisense *OsPPR1* exhibit abnormal chloroplast shape and reduced chlorophyll contents, leading to an albinism and lethal phenotypes (Gothandam et al., 2005). Subsequent research, employing forward or reverse genetics approaches, has identified numerous other *PPR* genes contributing to rice chloroplast development (**Table 1**; **Figure 3**).

WAL3, a PLS-type PPR protein, is crucial for rice chloroplast development. Its mutation causes an albino lethal phenotype by disrupting splicing of multiple group II introns, including *atpF*, *ndhA, petB*, *rps12*, and *rpl2*. This mutation affects chlorophyll synthesis and photosynthetic metabolic pathways (Lv et al., 2022). YLWS, a P-type PPR protein with 11 PPR motifs, directly binds to specific sites on *atpF*, *rpl2*, and *ndhA* pre-mRNAs, regulating their intron splicing efficiency. YLWS also impacts *ndhA*, *ndhB*, and *rps14* transcript editing. Disruption of *YLWS* leads to defective chloroplast development, characterized by vacuolated plastids and disorganized thylakoid membranes, resulting in the white-striped leaf phenotype (Lan et al., 2023). ALS3 regulates transcriptional levels of plastid translation machinery-associated genes and photosynthesis. Its mutation causes albino seedling lethality (Lin et al., 2015). OSOTP51, functionally conserved among higher plants, affects chloroplast *ycf3* mRNA intron splicing. *OSOTP51* mutation induces widespread changes in PSI structure and function and leads to severe photoinhibition, and albino phenotype (Ye et al., 2012).

WSL4, a P-family PPR protein, localizes to chloroplasts. Mutation of *WSL4* disrupts splicing of four group II introns (*ndhA*, *atpF*, *rpl2*, and *rps12*), resulting in white-striped leaves in rice seedlings (Wang et al., 2017). PGL12 is involved in *ndhA* splicing and 16S rRNA processing. The *pgl12* mutant exhibit yellow-green leaves, gradually becoming pale green as plants grow (Chen et al., 2019). OsPPR16, a chloroplast-targeted PLS-DYW subfamily protein with 14 PPR motifs, edits the chloroplast *rpoB* mRNA. Knockout of *OsPPR16* reduces *rpoB* accumulation and PEP-dependent gene expression, thereby leading to a pale phenotype (Huang et al., 2020). YSA, a P-type PPR protein with 16 tandem PPR motifs, is localized in chloroplasts. The *ysa* mutants shows albino leaves before the three-leaf stage, which gradually return to normal green by the six-leaf stage. Although chloroplast development is affected in *ysa* mutant, the main agronomic traits such as plant height, grain weight, and seed setting rate remain unaltered compared to WT. Therefore, in hybrid rice production, *ysa* mutants serve as a selective marker for effectively identifying and eliminating false hybrids (Su et al., 2012).

OsPPR4 regulates photosynthesis, chlorophyll, and chloroplast biosynthesis by directly binding to a specific sequence of chloroplast *rps12* intron 1, thereby affecting its splicing, and indirectly influencing the splicing of *rps12*, *ndhA*, *atpF*, and *petB* introns. The loss-of-function *osppr4* mutant exhibits an albino phenotype and fails to survive past the young seedling stage (Lee et al., 2019). OsPPR11, a P-type PPR protein, is responsible for splicing *ndhA* and *ycf3–1* introns. The *osppr11* mutant displays yellowing leaves and defective chloroplast development (Zhang et al., 2023). OspTAC2 plays a critical role in chloroplast development, with its mutation causes reduced chlorophyll content, electron transport, and photochemical reactions of photosynthesis. Its mutant exhibits albino seedlings after germination, with death occurring about two weeks later (Wang et al., 2016). OsSLC1, a member of the P subgroup of PPR proteins, is involved in splicing *rps*16 introns. The *slc1* mutant displayed chlorosis (Lv et al., 2020). *OsPPR6* encodes a plastid-localized PLS subfamily protein, which is involved in *ndhB* transcript editing and *ycf3* transcript splicing. The *osppr6* mutant exhibits early chloroplast developmental defects, albino leaves, and seedling death (Tang et al., 2017).

The chloroplast-targeted PPR proteins discussed earlier are involved in chloroplast development. Additionally, two mitochondria-targeted PPR proteins, MPR25 and OsPGL1, also play roles in this process. MPR25, a member of the PPR family’s E subgroup, participates in C-U RNA editing of *nad5* transcripts by directly interacting with the editing site. Mutation in *MPR25* prevents C-U RNA editing, resulting in pale-green leaves with reduced chlorophyll (Toda et al., 2012). OsPGL1, which targets both chloroplasts and mitochondria, edits *ndhD*-878 in chloroplasts and *ccmFc*-543 in mitochondria. It interacts with three OsMORFs (OsMORF2/8/9), which suggests involvement in RNA editing via an editosome. The loss-of-function in *ospgl1* leads to reduced chlorophyll content and defective chloroplast development, resulting in pale green leaves (Xiao et al., 2018b).

Under cold stress in rice, several PPR proteins, including TCD10, OsV4, ATP4, WSL5, CDE4, and DUA1, are essential for chloroplast development. *TCD10* and *OsV4* encode chloroplast-localized PPR proteins with 27 and 4 PPR motifs, respectively. Mutants of these genes exhibit reduced expression of chloroplast-associated genes, defective chloroplast development, and albino phenotypes under cold stress (Gong et al., 2014; Wu et al., 2016). ATP4, a PPR-SMR protein, is crucial for accumulating dicistronic *rpl16*-*rpl14* transcripts and the C-U editing of *rps8* transcripts. Loss of *ATP4* function results in a chlorotic phenotype (Zhang et al., 2020b). Both *wsl5* and *ced4* mutants showed leaf albino phenotypes at low temperatures. WSL5 primarily regulates chloroplast development by affecting the splicing of group II introns, *rpl2*, and *rps12* (Liu et al., 2018). CDE4, a P-type PPR protein, binds directly to pre-mRNA of *rpl2*, *ndhA*, and *ndhB* in chloroplasts, thereby regulating their intron splicing. It interacts physically with guanylate kinase V2. Overexpressing *V2* in *cde4* mutants restores group II intron splicing efficiency and mutant phenotype. Under cold stress, V2 likely stabilizes CDE4 protein, ensuring normal intron splicing that is necessary for chloroplast development (Liu et al., 2021). DUA1, localized in chloroplasts, interacts with RNA editing cofactor WSP1 and chloroplast sigma factor OsSIG1, thereby regulating editing efficiency of *rpoB*-C545, *rpoB*-C560, and *rps8*-C182 sites. Among which, DUA1 directly binds *rps8* transcripts. WSP1 enhances DUA1 protein stability under cold stress (Cui et al., 2019). Additionally, DUA1 interacts with a multiple organellar RNA editing factor OsMORF9, a determinant of chloroplast development (Zhang et al., 2021). The *dua1* mutants exhibited defective chloroplast development, chlorophyll biosynthesis, and albino phenotype at low temperatures (Cui et al., 2019; Du et al., 2021).

## 6 Conclusions and perspectives

In conclusion, PPR proteins play critical roles in fertility, biotic and abiotic stress responses, grain quality, and chloroplast development in rice (**Figure 4**). Research focusing on PPR protein function provides valuable gene resources for rice breeding. Among the 491 PPR proteins identified in rice, only ~9.7% (about 48) have been functionally characterized (**Figure 4**; **Table 1**), leaving the roles of the remaining PPR proteins unclear. It is imperative to comprehensively understand PPR protein functions. An effective strategy for elucidating PPR protein function is employing emerging genome-editing technologies like CRISPR/Cas9, which allow researchers to knockout candidate genes and determine their functions. Additionally, nearly half of the identified PPR proteins are implicated in chloroplast development. Mutations in these proteins lead to chlorophyll-deficient phenotypes and impair photosynthesis (**Figure 4**). Thus, investigating whether overexpression of specific *PPR* genes can enhance photosynthesis and rice yield is warranted. In rice, function losses of 5 PPR genes including *PPR405*, *PPR406*, *SOP10*, *OsSOAR1*, and *OsNBL3* improve the abilities to tolerant biotic and abiotic stress. Thus we can use the CRISPR/Cas9 technology to seek the PPR genes that can improve rice stress tolerance without yield loss.

PPR proteins, encoded by the nuclear genome, are targeted post-translationally to chloroplasts and mitochondria, where they participate in organelle RNA processing. Numerous studies indicate their role in regulating rice growth and development by editing, stabilizing, and splicing RNA transcripts in these organelles. Thus, identifying additional mitochondria and chloroplast RNA transcripts regulated by PPR proteins could serve as an ideal model for understanding interactions between organelle RNA and nuclear genes, thereby shedding light on signal transduction between the nucleus and cytoplasm. Furthermore, studying the expression of organelle genes regulated by PPR proteins can reveal key target genes influencing organelle development and agronomic traits in rice. For example, knocking out the rice mitochondrial gene *orf79* using mitoTALENs technology was found to successfully generate a new CMS line (Kazama et al., 2016).

Some PPR proteins have been observed to interact with OsMORFs, forming editosome complexes and participating in organelle RNA editing processes. However, to date, most identified PPR proteins function independently, without interactions with other proteins. Consequently, it is necessary to investigate whether PPR proteins interact with or form spliceosome complexes with other proteins to regulate organelle RNA processing in rice. In rice, most PPR proteins are targeted in mitochondria or chloroplast, whereas several PPR proteins including OsPPR676, OsCPPR1, WSL, FGR1, and FLO14 are targeted in nucleus and cytoplasm. Are there any differences in structure and regulation mechanism between these two types of proteins that warrant further study. Furthermore, it is intriguing that different types of PPR proteins are sometimes required for the expression of the same organellar genes. Therefore, this raises the question that whether these PPR proteins interact with or regulate each other. Thus, focusing future research on these aspects will be instrumental in unraveling the regulatory mechanisms of PPR proteins.

## Author contributions

LM: Writing – original draft. MD: Writing – original draft. TZ: Writing – original draft. GL: Writing – original draft. YD: Writing – original draft. QZ: Writing – review & editing.
